# Distal radial fractures in Parkinson’s disease: 3179 cases from the Swedish Fracture Register

**DOI:** 10.1177/17531934251349674

**Published:** 2025-06-14

**Authors:** Marcus Sagerfors, Theres Dyrvén, Tahir Taj, Evelina Pantzar Castilla, Peter Wildeman

**Affiliations:** 1 Department of Orthopedics and Hand Surgery, Faculty of Medicine and Health, Örebro University, Sweden; 2 Department of Clinical Epidemiology and Biostatistics, School of Medical Sciences, Örebro University, Sweden

**Keywords:** Distal radius fracture, epidemiology, mortality, Parkinson’s disease, patient-reported outcome

## Abstract

This study aimed to investigate epidemiology, injury characteristics, treatment, mortality and patient-reported outcome measures in patients with a distal radial fracture and Parkinson’s disease. The patient population was obtained from the Swedish Fracture Register, and patients with Parkinson’s disease were identified by data from the National Board of Health and Welfare. A total of 3179 cases were identified and matched with controls. Most fractures took place in the patient’s home after a simple fall and were managed non-operatively. An anterior locking plate was the most common surgical intervention. The 1 year mortality was significantly higher among men with Parkinson’s disease than in controls. Patient-reported outcome measures at the 1 year follow-up deteriorated significantly more for patients with Parkinson’s disease than in controls, and patients with Parkinson’s disease also experienced more problems with reoperations, pain, mobility and carrying out their usual activities.

**Level of evidence:** III

## Introduction

Parkinson’s disease (PD) is a common, chronic and progressive neurological disorder characterized by rigidity, unsteady gait and postural instability (Cheng et al., 2014; Lindholm et al., 2024). The disease is associated with increased morbidity, functional dependence and a shorter life span, as well as economic consequences such as loss of earnings and cost of care (Dorsey et al., 2018a). Patients with PD have an almost twofold increased risk of fractures compared with the general population, not only because of an increased risk of falls but also related to advanced age, comorbidities and osteoporosis (Coomber et al., 2017). In addition, medications like monoamine oxidase-B inhibitors, high-dose antipsychotics, and selective serotonin receptor inhibitors substantially increase the risk of osteoporotic fractures (Pouwels et al., 2013). From 1990 to 2015, the number of people with PD more than doubled (Dorsey et al., 2018b). Patients with PD are known to have inferior results, more frequent complications, and an increased rate of treatment failure after orthopaedic trauma (Eventov et al., 1983; Zuckerman, 2009). Studies on distal radial fractures (DRFs) in patients with PD are sparse but indicate high rates of complications and treatment failures (Chou et al., 2020; Logli and Rizzo, 2022).

The Swedish Fracture Register (SFR) is a nationwide register that has prospectively collected data on orthopaedic fractures since 2011. The aim of this study was to assess epidemiology, injury characteristics, treatment, reoperations, mortality and patient-reported outcome in patients with Parkinson’s disease who sustained a distal radial fracture using data from the Swedish Fracture Registry.

## Methods

The study included all non-pathological DRFs registered in the SFR between 1 January 2012 and 30 December 2022 in patients over 18 years of age who had been prescribed Parkinson’s medication before the DRF. Registration of DRFs in the SFR is done prospectively by the attending surgeon or junior doctor at each affiliated department of each affiliated hospital, using a web-based platform (Möller et al., 2022). Only fractures occurring in Sweden and in patients with a Swedish personal identity number are registered. At the beginning of 2015, half of the departments treating fractures in Sweden had joined the SFR, and by 2021 all orthopaedic departments in Sweden treating DRFs had joined (Bergvall et al., 2021; Juto et al., 2016; Marsh et al., 2007; Wennergren et al., 2016, 2017). A manual with schematic images of the different fracture groups is used in the online classification. Injury location is classified as the patient’s residence (including institutional housing), on a street or road, in a public space or at an unspecified place.

Patient-reported outcome measures (PROMs) are also included in the SFR. Questionnaires are sent to the patient a short time after the fracture, and the patient is asked to report their health status during the week before the fracture occurred (recall technique). A reminder is sent after 4 weeks. The 1 year follow-up questionnaires are sent only to those patients who completed the initial questionnaires. They include the validated Swedish translations of the EuroQuol Five Dimension Three Level (EQ-5D-3L) PROM, the EQ visual analogue scale (EQ-VAS) and the Short Musculoskeletal Function Assessment (SMFA) (Ponzer et al., 2003; Rundgren et al., 2018).

The EQ-5D-3L is a common generic questionnaire measuring health-related quality of life for a range of conditions and treatments (Devlin and Brooks, 2017). Respondents assess their health status across five dimensions: mobility, self-care, usual activities, pain/discomfort and anxiety/depression, with each dimension measured by a single question. Their responses can be summarized as an index value calculated using an experience-based value set (Burström et al., 2020) and anchored at 1 for full health and 0 for death. The EQ-VAS range is 0–100, with 100 representing the best imaginable health state and 0 the worst imaginable health state.

The SMFA is designed to estimate the functional status of patients with a wide range of musculoskeletal injuries and disorders. The Swedish version has been shown to be reliable and sensitive to changes over time (Ponzer et al., 2003). It has two parts: a dysfunction index with 34 items, and a bother index with 12 items. The dysfunction items are divided into four categories: daily activities, emotional status, function of the arm and hand and mobility. A formula gives a final score ranging from 0 to 100, where higher scores indicate poorer function.

Mortality is also included in the SFR and is updated daily via a link to the Swedish Tax Agency. For this study, 1 year mortality was calculated.

Patients with PD were identified by cross-matching all DRF cases from the SFR with data on medication for PD from the National Prescribed Drug Register administered by the National Board of Health and Welfare. Individuals were classified as having PD if their prescription records showed that they had been prescribed at least one medication from the N04 ATC code group, which includes drugs specifically used for Parkinson’s treatment. This classification ensured a systematic identification of patients receiving pharmacological treatment for PD based on prescription records.

### Selection of controls

**Figure 1.**
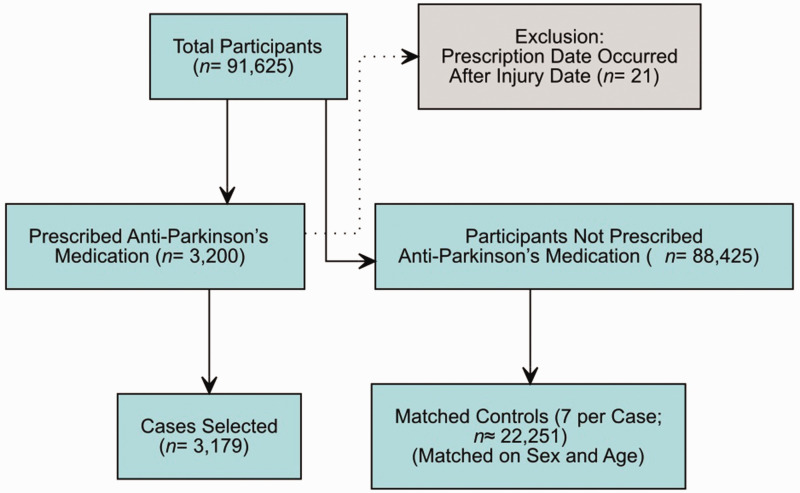
Flow chart showing inclusion and exclusion of participants.

Controls were selected using a matched case–control design. For each patient with PD (case) who sustained a DRF, seven unique controls were selected from the pool of participants from the SFR without PD, while ensuring that no control was selected more than once. Matching was based on key demographic characteristics, specifically age and sex, to ensure that cases and controls were comparable. Figure 1 shows the flowchart detailing the selection process.

### Statistical methods

Descriptive statistics were calculated separately for cases and controls. Continuous variables (e.g. age) are reported as the mean (standard deviation) and categorical variables (e.g. sex, injury cause, fracture classification) are presented as counts (percentages). To compare outcomes between matched cases and controls, conditional logistic regression was used for binary outcomes, with the matching identifier included to account for the paired design. For continuous outcomes measured at multiple time points (e.g. preoperative and postoperative measures), paired *t-*tests were employed.

For each category regarding baseline demographics and injury characteristics, conditional logistic regression (accounting for matching) was used to compare cases with controls. For each category of injury locations, a binary conditional logistic regression (using the matching identifier) was used to compare the odds of being a case among those with that injury versus all other injuries. The resulting odds ratio and its 95% confidence interval reflect the relative odds of being a case, while the *p*-value (from the *Z*-test) indicates whether the difference is statistically significant (Table 1).

**Table 1.** Baseline demographic and injury characteristics by AO fracture type A–C in patients with Parkinson’s disease (cases) and matched controls.

	Type A(extra-articular)		Type B(partial articular)		Type C(complete articular)		
	Cases	Controls	Cases	Controls	Cases	Controls	*p*-value
Age, mean (SD)	72.5 (13)	72.5 (13)	69.4 (15)	70.0 (14)	70.8 (12)	70.6 (13)	**<0.001**
Sex							**<0.001**
Female	1762 (86)	11914 (84)	248 (71)	1775 (73)	586 (81)	4547 (82)	
Male	311 (15)	2235 (16)	101 (29)	651(27)	151 (19)	1031 (19)	
Cause of injury							0.032
Fall from a height	75 (3.5)	407 (2.9)	11 (3.0)	62 (2.6)	32 (4.2)	171 (3.1)	
Other accidental injury	1 (0.0)	19 (0.1)	0 (0.0)	3 (0.1)	0 (0.0)	0 (0.0)	
Other external cause	9 (0.5)	51 (0.4)	2 (0.5)	19 (0.8)	4 (0.5)	19 (0.3)	
Simple fall	1730 (84)	11805 (83)	268 (76)	1865 (77)	580 (79)	4366 (78)	
Traffic accident	4 (0.2)	15 (0.1)	0 (0.0)	5 (0.2)	2 (0.2)	18 (0.3)	
Assault, self-harm, or unspecified	1 (0.0)	0 (0.0)	0 (0.0)	0 (0.0)	0 (0.0)	0 (0.0)	
Unknown	253 (12)	1852 (13)	68 (20)	472 (20)	119 (16)	1004 (18)	

Results are shown as *n* (%) for categorical variables. Statistically significant *p*-values shown in bold.

Treatment categories were derived from the original treatment codes using a predefined mapping. Data are presented as *n* (%), with percentages indicating the proportion of participants within each group (cases or controls). For each treatment category, a conditional logistic regression analysis was performed using a binary indicator (1 = participant received that treatment; 0 = did not) and including the matching identifier as a stratification variable (Table 2). One-year mortality for patients with Parkinson’s disease and controls were assessed per age group and sex (Table 3).

**Table 2.** Type of treatment for distal radius fractures (DRFs) in patients with Parkinson’s disease (cases) vs. controls.

Treatment	Cases*n = *3179	Controls*n = *22215	*p*-value
Non-surgical immobilization	2195 (69%)	15332 (69%)	0.871
Volar plate	813 (26%)	6033 (27%)	0.067
Pins or K-wires	21 (0.7%)	78 (0.4%)	**0.010**
External fixation	2 (0.1%)	38 (0.2%)	0.168
Dorsal plate	11 (0.3%)	24 (0.1%)	**0.001**
Other treatments	137 (4.3%)	746 (3.4%)	**0.006**

Statistically significant *p*-values shown in bold.

**Table 3.** One-year mortality for patients with Parkinson’s disease (cases) and controls per sex and age group.

		Cases			Controls	
	Total (*n = *3179)	Deaths at 1 year (*n = *216)	Mortality rate (%)	Total (*n = *22,215)	Deaths at 1 year (*n = *1057)	Mortality rate (%)	*p*-value
Sex							
Female	2614	152	5.8	18298	821	4.5	Ref
Male	565	52	9.2	3953	237	6.0	**0.002**
Age group							
<50	163	3	1.8	1141	2	0.2	Ref
50–59	411	6	1.5	2877	17	0.6	0.741
60–69	654	19	2.9	4578	52	1.1	0.456
70–79	987	50	5.1	6909	140	2.0	0.081
80–89	755	83	11	5285	480	9.1	**0.002**
90+	209	43	21	1461	367	25	**<0.001**

Results are presented as *n* (%), or as percentage for the 1 year mortality rate (calculated as [total deaths/total patients] × 100). Statistically significant *p*-values shown in bold.

For the EQ-5D scores, differences between cases and controls were assessed using conditional logistic regression (accounting for the matching) for each domain; a *p*-value < 0.05 indicates a statistically significant difference (Table 4). Regarding the 1 year outcome, data are shown as mean (standard deviation). For each outcome, paired comparisons between cases and controls were performed using paired *t-*tests (accounting for the matching design) (Table 5).

**Table 4.** Distribution of baseline EuroQuol- 5 Dimension scores by category in patients with Parkinson’s disease (cases) and matched controls.

	Cases			Controls			
Domain	No problems	Moderate problems	Severe problems	No problems	Moderate problems	Severe problems	*p*-value
Anxiety	573 (16%)	290 (8.3%)	32 (0.9%)	4540 (20%)	1128 (5.1%)	100 (0.4%)	**<0.001**
Pain	334 (9.5%)	473 (14%)	84 (2.4%)	3319 (15%)	2247 (10%)	191 (0.9%)	**<0.001**
Mobility	593 (17%)	295 (8.4%)	2 (0.1%)	4666 (21%)	1083 (4.9%)	15 (0.1%)	**<0.001**
Self-care	708 (20%)	164 (4.7%)	22 (0.6%)	5088 (23%)	562 (2.5%)	128 (0.6%)	**<0.001**
Usual activities	629 (18%)	186 (5.3%)	78 (2.2%)	4784 (22%)	673 (3.0%)	303 (1.4%)	**<0.001**

Data are shown as *n* (%). Statistically significant *p*-values shown in bold.

**Table 5.** One-year outcomes following distal radius fracture in patients with Parkinson’s disease (cases) and matched controls.

	Baseline			One year after fracture			Change		
	Cases	Controls	*p*-value	Cases	Controls	*p*-value	Cases	Controls	*p*-value
EQ-5D index	0.85 (0.13)	0.90 (0.09)	**<0.001**	0.83 (0.14)	0.90 (0.10)	**<0.001**	−0.02 (0.12)	−0.01 (0.08)	0.2812
EQ-5D VAS	71.32 (22.62)	78.74 (14.83)	**<0.001**	70.95 (21.59)	78.64 (16.15)	**<0.001**	−0.96 (22.90)	−0.86 (15.75)	0.5597
SMFA arm/hand	15.12 (21.58)	9.00 (13.14)	**<0.001**	19.18 (21.27)	12.44 (14.14)	**<0.001**	6.84 (18.53)	4.40 (11.57)	**<0.001**
SMFA bother	18.81 (19.62)	11.29 (12.06)	**<0.001**	22.24 (21.61)	14.59 (13.17)	**<0.001**	6.10 (18.11)	4.24 (11.57)	0.0529

Data are shown as mean (SD). EQ-5D, EuroQuol-5 Dimension; VAS, visual analogue scale; SMFA, Short Musculoskeletal Function Assessment. Statistically significant *p*-values shown in bold.

Normality of continuous variables was assessed using the Shapiro–Wilk test. As the data were approximately normally distributed, parametric methods (e.g. paired *t*-tests) were used for comparisons. A *p*-value < 0.05 was considered statistically significant throughout the analyses.

## Results

### Epidemiology

Of the 91 625 DRF cases registered during the relevant period, 3179 cases with PD were identified, comprising 2614 women and 565 men (Figure 1). Regarding fracture type, 2073 fractures were AO type A, 349 were type B, 737 were type C and 20 were classified as ‘other’. The most common injury mechanism was a simple fall, both for PD cases and for controls. Most of the PD cases and most of the controls were female (Table 1). February was the month with the highest number of DRFs, and July was the month with the lowest. The most common reported location for both PD cases and controls was their residence (40 and 35% respectively; *p* < 0.05), followed by the street or road (9.1 and 11%).

Most of the PD cases and the controls were treated with non-surgical immobilization. An anterior plate was the most common surgical intervention reported for both groups. The most common treatment methods did not differ between PD cases and controls (Table 2). Patients treated with an anterior plate were operated by a resident in orthopaedic surgery in 23% of the PD cases (23% for controls), a specialist in orthopaedic surgery in 37% of the PD cases (37% for controls) and an orthopaedic trauma surgeon in 27% of the PD cases (27% for controls). The frequency of open fractures was 0.6% for the PD cases and 0.5% for the controls, and the frequency of high-energy trauma was 2.4% for both cases and controls.

#### Mortality

The 1 year mortality rates in the various groups are shown in Table 3. There was a significant difference in 1 year mortality between the PD cases and controls (*p* = 0.002) when all the men were analysed together, but when analysed by age group there was only a significant difference in PD patients aged over 80 years.

#### Patient-reported outcome measures

There was a significant difference between PD cases and matched controls at baseline. One year after the fracture, the proportion of patients who reported severe problems with mobility and self-care was considerably higher than at baseline, while the proportion of patients reporting moderate problems with usual activities was somewhat higher than at baseline (Table 4). The EQ-5D index, but not the EQ-5D VAS, had deteriorated significantly at the 1 year follow-up. Both SMFA indices, the arm/hand index and the bother index had deteriorated significantly at the 1 year follow-up (Table 5). The frequency of reoperations within 30 days owing to failed operative fixation was 0.4% for the cases and 0.1% for the controls. The response rate regarding PROMs was somewhat higher for the controls compared with the PD patients (29 vs. 26%).

## Discussion

The findings of this study indicate that male PD patients with a DRF have a 1 year mortality significantly higher than that for controls. A Swedish study on patients over 80 years with a DRF found a 1 year mortality of 5% (Arvidsson et al., 2024); the 1 year mortality among the PD patients with a DRF in the present study was higher than this, even though the mean age of the presented patients was lower. In contrast, a previous study on very elderly patients (age > 80 years) with a DRF using data from the SFR found a 1 year mortality of 11.8% (Sagerfors et al., 2022). The 1 year mortality in the current study was considerably higher than previously reported for a DRF, but this is probably related to comorbidities and frailty in this patient group (Hommel et al., 2022).

The most common place of injury was the patient’s home following a simple fall, which is in line with the findings of another study on DRF epidemiology in Sweden (Rundgren et al., 2020). Compared with the controls, significantly more fractures occurred at home or in institutional housing in the patients with PD, probably reflecting their sedentary lifestyle (Khan et al., 2024).

The relative incidence of AO type B fractures was somewhat lower than in another study (Rundgren et al., 2020). One explanation for this difference could be that patients with PD possibly have an inferior bone quality compared with the general population, resulting in a lower incidence of shearing type B partial articular fractures and instead a higher relative incidence of type A and C fractures (Falaschi et al., 2021). The DRF classification in the SFR has a moderate accuracy, as shown previously (Bergvall et al., 2021; Plant et al., 2015). The fractures in this study were classified by the treating physician, reflecting real-life conditions where classifications are made by orthopaedic surgeons with different levels of experience.

October and February were the months with the highest frequency of DRFs. This is in line with a previous study which found that the incidence of DRF was twice as high in January compared with May, probably related to ice and slippery streets during the winter in Sweden (Rundgren et al., 2020).

In the PD cohort, 26% of the DRFs were treated surgically with an anterior plate. This proportion is somewhat higher compared with a previous national register study, which showed that in 2010 the proportion of surgically managed DRFs was 20% (Mellstrand-Navarro et al., 2014). A likely reason for the higher frequency is that anterior plating has increased in popularity over time (Hevonkorpi et al., 2018).

PROMs worsened significantly in patient with PD compared with controls. This may reflect complications and a poor outcome, but may also reflect general problems with pain, mobility and arm/hand function in persons with PD (Lees et al., 2009). Problems with reported anxiety were significantly higher in PD patients than in controls. Depression and anxiety are present in 35% of patients with PD, and have also been associated with dementia (Hayes, 2019). Anxiety has previously been linked to inferior PROMs following a DRF, and this finding could partially explain the inferior PROMs regarding hand function in PD patients compared with controls (Jakobsson et al., 2024). One confounder in using a generic PROM like the EQ-5D-3L may be that DRF patients with PD are likely to have limitations regarding mobility and self-care (Hanff et al., 2024).

The EQ-5D-3L has been shown to have an acceptable to good responsiveness in patients with a DRF (Rundgren et al., 2018). The fact that the EQ-5D-3L and SMFA are not PROMs designed specifically for upper extremity disorders may limit their ability to identify a deterioration in wrist function, compared to more disease-specific upper-extremity PROMs like the Disabilities of the Arm, Shoulder and Hand and Patient-Rated Wrist Evaluation. Parkinson’s disease is also a chronic progressive disorder and some of the deterioration in PROMs may be attributable to the disease itself (Lees et al., 2009). However, both the EQ-5D-3L and the SMFA reflect the patient’s general well-being, which is arguably an important outcome measure but one that is difficult to interpret in the presence of PD, which has general effects on body function.

The frequencies of open fractures and high-energy trauma were low. This contrasts with the rate of 2.5% open fractures previously reported for very elderly patients (80+ years) with DRFs in the SFR, possibly related to the ageing process altering the mechanical properties of the skin and its elastin and collagen organization (Sagerfors et al., 2022).

Treatment was given based on local traditions and personal preferences rather than common guidelines. There is a considerable variation in the operative management of DRF between healthcare regions (Saving et al., 2018). The experience levels of the operating surgeons were similar for the PD cases and the controls; most of the operations were done by specialists in orthopaedic surgery. The frequency of reoperations because of failed operative fixation was low for both the PD cases and the controls, but still substantially higher for the PD cases in comparison. There are indications that the completeness of registrations of reoperations in the SFR is suboptimal, but this is likely to be the case both for PD cases and controls (Kapetanovic, 2015).

At the end of 2017, the SFR covered more than 80% of the population in Sweden, and so the data can be expected to give a good representation of DRF treatment in Sweden (Rundgren et al., 2020). Completeness of fracture data has been assessed for the SFR, and the register is considered a complete and accurate source of information (Bergdahl et al., 2021). The risk of bias owing to varying local treatment traditions and sociodemographic and epidemiological differences is somewhat ameliorated by the large number of patients with prospectively registered data on a national level. Matching cases and controls by age and sex minimized confounding by these factors, thereby allowing more reliable comparisons between the two groups. Data were registered in a pre-specified way, and to our knowledge the study is unique in size and detail regarding DRFs in patients with PD. Coverage improved during the study period, but the lack of full national coverage is a limitation. A limitation of all register studies of this type is the response rate, which to some degree was mitigated by the relatively large number of fractures in the study. In addition, data from a previous study indicate that the PROMs of non-responders are similar to those of initial responders (Juto et al., 2017). This study is also limited by the fact that radiographic outcome 1 year after the fracture was not available. However, the correlation between radiographic outcome and PROMs after a DRF is unclear (Clement et al., 2014; Goldfarb et al., 2006; Grewal and MacDermid, 2007; Lundqvist et al., 2022). Another limitation is recall bias. At the time of inclusion patients were asked to fill out the questionnaires for their pre-injury status as it was in the week before the injury, which may overestimate the change in PROMs (Scholten et al., 2017). In addition, dementia is associated with PD and could affect recall ability (Lees et al., 2009).

In conclusion, the findings of this study indicate that in comparison with controls, patients with PD and a DRF have a higher reoperation frequency, experience considerably larger deterioration of PROMs and also experience more problems with pain, mobility and in their usual activities.

## Data Availability

The datasets used and/or analysed during the current study are available from the corresponding author on reasonable request.
